# Removing atherosclerotic plaque created using high cholesterol diet in rabbit using ultrasound

**DOI:** 10.1186/s40349-015-0025-8

**Published:** 2015-01-29

**Authors:** Christakis Damianou, Christos Christofi, Nicos Mylonas

**Affiliations:** Electrical Engineering Department, Cyprus University of Technology, Limassol, Cyprus; R&D Department, MEDSONIC, LTD, Limassol, Cyprus; Computer Science Department, Frederick Research Center, Limassol, Cyprus

**Keywords:** Ultrasound, Atherosclerotic, Plaque, Pulse

## Abstract

**Background:**

The aim of the proposed study was to conduct a feasibility study using a flat rectangular (3 × 10 mm^2^) transducer operating at 5 MHz for removing atherosclerotic plaque in an *in vivo* model. The proposed method can be used in the future for treating atherosclerotic plaques in humans.

**Methods and results:**

The plaque in the rabbits was created using high cholesterol diet for 4 months. The amount of plaque removed was studied as a function of intensity, with a fixed pulse repetition frequency (PRF), and duty factor (DF).

**Conclusions:**

The amount of plaque removed is directly related to the acoustic intensity. It was found that the presence of bubbles accelerates the removal of plaque. In order to ensure that pure mechanical mode ultrasound was used, the intensity used does not produce temperatures that exceed 1°C.

## Introduction

Atherosclerosis is a condition in which fatty material collects along the walls of arteries. This fatty material thickens and may eventually block the arteries [1]. The plaque is composed of distinctly morphological features including a fibrous cap comprised of smooth muscle cells, fibrotic tissue, and lipid core containing fat-laden macrophages and extracellular lipids [1]. In the advanced stage, atherosclerotic plaque contains large amounts of calcium salt [2], which significantly increases the mechanical properties of the plaque. Histology studies have also led to the recognition that plaque structure influences the risk of plaque rupture. Specifically, a plaque with a thin fibrous cap and a large lipid core is more prone to rupture [3].

Lifestyle changes, such as eating a healthy diet and exercising, are often the best treatment for atherosclerosis. But sometimes, medication or surgical procedures may be recommended as well [4]. Atherosclerosis treatment may require special surgical procedures such as Balloon Angioplasty [5-7], balloon angioplasty and stenting [8,9], cutting balloon [10-12], atherectomy [13,14], and surgical bypass [15,16], to open an artery and improve blood flow. The main treatment for the carotid artery is endarterectomy [17-19].

Recently therapeutic ultrasound has been employed in the hospitals for many applications. Therapeutic ultrasound was utilized on prostate carcinoma in rats, where it was proven that thermal ultrasound has the potential to treat small localized prostate cancer lesions [20]. Clinical trials utilizing thermal ultrasound and ultrasound imaging were published thereafter [21,22]. The two dominant clinically available systems using transrectal HIFU are Ablatherm HIFU (Technomed International, Lyon, France) and HIFU Sonablate 500 (Focal Surgery, Milpitas, CA now SonaCare Medical, Charlotte, NC, USA). Recent advances of the transrectal device includes a phased-array probe for more efficient treatment of the prostate [23]. Another clinical success that employs therapeutic ultrasound with magnetic imaging guidance (MRI) is the technology introduced by the Israeli company InSightec [24]. This technology resulted in the first commercial system for the treatment of uterine fibroids (using also thermal ultrasound), which received approval by the Food and Drug Administration (FDA) in 2004. This system is incorporated in the table of a General Electric MRI scanner. The system initially was approved for the treatment of various gynecological tumors [25-28]. An endorectal thermal ultrasound system produced by the same company has been utilized recently for the treatment of prostate cancer [29,30]. The same company utilized thermal ultrasound for pain palliation of bone metastases [31-33]. Finally, InSightec developed a transcranial MRI-guided HIFU system for the non-invasive treatment of various brain diseases [34] such as brain cancer, and Parkinson’s disease (thermal ultrasound), and stroke (mechanical ultrasound using microbubbles). Philips Healthcare, Netherland showed interest in this technology recently and, as a result the MRI guide HIFU system, Sonalleve was developed as a commercial product [35] for the treatment of uterine fibroids and bone palliation using thermal ultrasound. This system is incorporated in the table of a Philips MRI scanner. Besides the two systems providing therapy for prostate diseases, there is another technology from China [36-39] that establishes extracorporeal thermal ablation for various organ using focused ultrasound and ultrasonic imaging (liver, breast, kidney, osteosarcoma, and pancreas).

Recently at the University of Minnesota, researchers attempted to treat atherosclerosis with noninvasive method such as HIFU [40]. The University of Minnesota recently developed a new HIFU technology that performs noninvasive, real-time ultrasonic imaging and localized treatment using thermal ultrasound.

In this paper, pulsed ultrasound was utilized for the first time for removing atherosclerotic plaque in an *in vivo* rabbit model. In order to ensure that pure mechanical mode ultrasound was used, the protocols were designed so that the temperature does not exceed 1°C. However, to our knowledge, no prior studies have been reported on ultrasound ablation of atherosclerotic plaque using pulsed ultrasound.

This paper describes a feasibility study that was carried out, in order to investigate the effectiveness of a therapeutic protocol in removing atherosclerotic plaque through pulsed ultrasound using a planar unfocused transducer operating at 5.3 MHz. An existing good model for developing atherosclerotic plaque [41,42] was used. In this animal model, high cholesterol diet is administered to rabbits. As a result, atherosclerotic plaque is formed in various arteries of the rabbit (aorta, abdominal, and carotid). The efficacy of pulsed ultrasound was monitored with ultrasonic imaging. The growth of the plaque was evaluated with histology of the arteries. Although the aorta is the biggest artery with size comparable to important arteries of humans (for example coronary), we have chosen to use the carotid artery for therapy, mainly to avoid breathing and because access to this artery with ultrasound is easy.

The main ultrasonic parameter evaluated for its effect on the treatment was the intensity. The duty factor (DF) was varied in just one experiment to show that there is limit on the value of DF to be used, since high DF may cause thermal effects. Thus, the main therapy parameter used in this study was the acoustic intensity.

## Materials and methods

### Experimental setup

**Figure 1:**
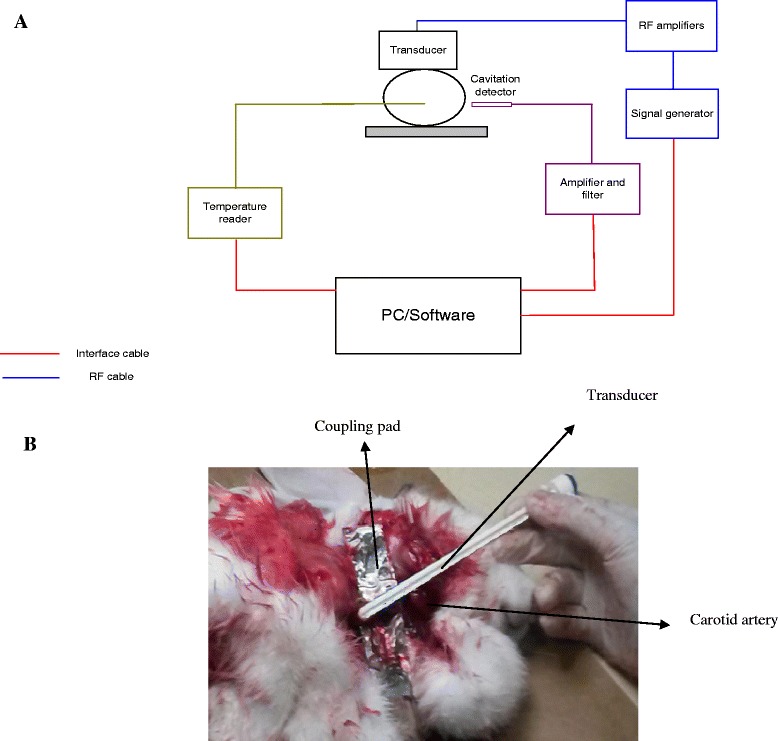
**System diagram and coupling to animal.** Diagram of the pulsed high intensity ultrasound system which includes a generator/amplifier, a temperature reader, and a passive cavitation detector; **(A)** and coupling of the transducer to the carotid artery **(B)**.

Figure 1A shows the schematic diagram of the ultrasound system. The apparatus is divided into two subsystems: (1) ultrasound generation, and (2) passive cavitation detection (a hydrophone was employed to monitor cavitation activity). A 5.3-MHz sinusoidal input was generated by a function generator (Agilent 33120 15 MHz Function/Arbitrary Waveform Generator, Englewood, CO, USA). The electrical signal is amplified by an RF amplifier (75 W, AR, Souderton, PA, USA), and then delivered through an impedance matching network designed exclusively for this particular transducer. Figure 1B shows the coupling of the transducer to the rabbit carotid artery.

**Figure 2:**
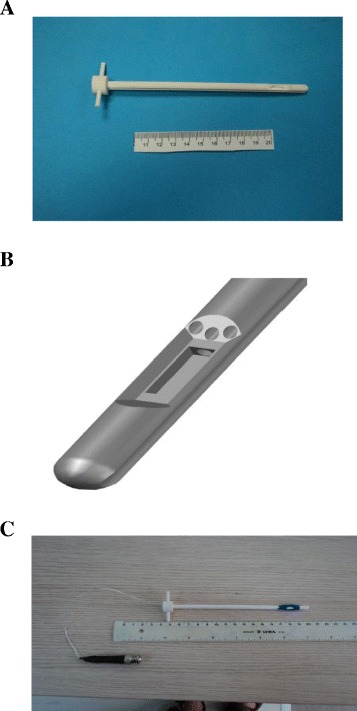
**Transducer design.** Transducer holder manufactured using ABS. This structure contains two inlets for transducer cooler and two inlets for the transducer wires **(A)**. Drawing of the transducer holder indicating the water inlets and wiring inlets **(B)**. Final assembly of the transducer inside the plastic holder **(C).**

The active size of the transducer is 3 × 10 mm^2^. The transducer material is P762-type PZT piezoceramic (Quartz & Silice, Nemours, France) with air backing, operating at 5.3 MHz. Figure 2A shows the transducer holder manufactured using acrylonitrile butadiene styrene (ABS). The ABS parts of the robot were designed using computer-aided design (CAD) software (Microstation V8, Bentley Systems, Inc., Exton, PA, USA). The files were then exported to computer-aided manufacturing (CAM) software (Insight V. 6.4.1, Stratasys Inc., Eden Prairie, Minnesota, USA). The files were sent to a 3D printer (FDM400, Stratasys, 7665 Commerce Way, Eden Prairie, MN, USA) for production. This structure contains two inlets for transducer cooling and two inlets for transducer wiring. Figure 2B shows the drawing of the transducer holder indicating the water inlets and wiring inlets. Figure 2C shows the final assembly of the transducer inside the plastic holder. Because in future clinical trials the transducer will be incorporated in a catheter that will be guided through arteries (1–3 mm wide), the transducer element must be as compact as possible. Since the catheter will be inserted in the body, after the treatment the catheter will be destroyed (therefore it is considered consumable).

Using the acoustic balance technique [43], the electroacoustic efficiency of the applicator was measured (55% at 5.3 MHz). The external face of the transducer was cooled by a continuous flow of degassed water circulating the length of the transducer. The water cooling circuit was maintained at 15°C and was driven by a Masterflex peristaltic pump (Cole Parmer Instrument Co., Chicago, IL, USA) at a flow of 0.15 L/min.

### Sonication parameters

The spatial-average pulse-average (SAPA) intensity was estimated, by dividing the power with the surface area of the transducer. The PRF used was 100 Hz. The duty factor varied from 10% to 40%. The sonication duration was 5 min because the ultrasound bubbles injected in the circulation system of the rabbit last for 5 min. Several 5-min sessions were used to effectively remove all the plaque desired.

### Cavitation detection

The cavitation activity was monitored using a hydrophone (Specialty Eng. Associates, Irvine, CA, USA). The signal from the hydrophone was fed to a custom-made amplifier (×20 amplification), and was high-pass-filtered using a custom-made filter. Fast Fourier transform (FFT) spectrums of the acoustic emission signals were acquired using a PC-based interface card (Gage, Lockport, NY, USA). The hydrophone was aligned perpendicular to the sample under investigation.

### Animals and diet

Totally 17 New Zealand rabbits (3.8–4 kg) were used during the experiments. The rabbits were divided randomly into two groups. The animals of group A (*n* = 2) were fed with normal chow. In group B (*n* = 15) the animals were fed with 2% high cholesterol diet (T2030, Harlan laboratories SRL, Udine, Italy). Twelve (12) rabbits in this group B were sacrificed without any treatment. These rabbits were used as a reference for evaluating the reduction of artery at the first month (*n* = 3), second month (*n* = 3), third month (*n* = 3), and fourth month (*n* = 3). Three (*n* = 3) rabbits were treated with ultrasound and microbubbles. Animals going through the high cholesterol diet were sacrificed to a maximum of 4 months, because severe side effects appeared (hypercholesteremic side effects such as weight loss, appetite loss, and jaundice).

### *In vivo* experiments

The rabbits were anaesthetized using a mixture of 500 mg of ketamine (100 mg/mL, Aveco, Ford Dodge, IA, USA), 160 mg of xylazine (20 mg/mL, Loyd Laboratories, Shenandoah, IA, USA), and 20 mg of acepromazine (10 mg/mL, Aveco, Ford Dodge, IA) at a dose of 1 mL/kg. The animal experiments protocol was approved by the national body in Cyprus responsible for animal studies (Ministry of Agriculture, Animal Services).

### Statistical analysis

Statistical analysis was performed using the software SIMA. Paired t-test analyses were used to compare lumen area reduction with and without high cholesterol. Correlation analyses were performed using linear regression analysis with 95% confidence intervals (*p* = 0.05). Same analysis was performed when assessing the effect of ultrasonic intensity on the plaque reduction.

### Ultrasound bubbles

Prior to the application of ultrasound a bolus of an ultrasound contrast agent (SonoVue; Bracco SpA, Milan, Italy) was injected intravenously through the ear vein at a dose of 0.02 mL/kg.

### Hematoxylin and eosin (ΗΕ) staining

By the end of the experiments the rabbits were sacrificed and transcardially perfused with 60 mL phosphate-buffered saline and 120 mL 4% paraformaldehyde. The artery was then soaked in paraformaldehyde for 24 h. Hematoxylin and eosin staining was performed on paraffin-embedded artery with a slice thickness of 10 μm for histologic examinations.

### Ultrasonic imaging

An ultrasonic system (Philips HD7 series Ultrasound Systems, Philips and Neusoft Medical Systems Co. Ltd, Shenyang, China) was used to monitor the plaque removal during ultrasound therapy using a 12-MHz probe dedicated for small structures.

### Temperature measurement

A data acquisition board (6251 DAQ, National Instruments, TX, USA) was used to measure the temperature in the artery. An analogue input of the board is used to capture the temperature. An Omega (M2813-1205, OMEGA Engineering, Inc. Stamford, CT, USA) voltage-to-temperature converter was used to measure temperature using a software written in MatLab (The Mathworks Inc., Natick, MA, USA). A thermocouple (Omega Engineering) was placed in the carotid artery to measure temperature elevation at the surface of the transducer since at that point maximum temperature is achieved. The size of the thermocouple was chosen to be 50 μm, so that the interaction with the beam of ultrasound is minimized.

## Results

**Figure 3:**
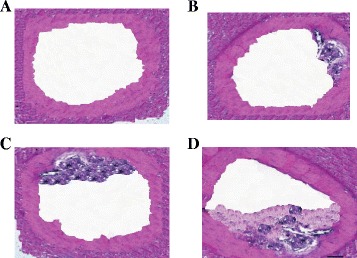
**Photos of the rabbit aorta with HE staining indicating the growth of the plaque within the artery.** 0 months **(A)**, 1 month **(B)**, 2 months **(C)**, and 3 months **(D)**.

Figure 3 shows photos of the rabbit aorta with HE staining indicating the growth of the plaque within the artery: A) 0 months, B) 1 month, C) 2 months, and D) 3 months. In order to acquire histology for a specific rabbit, the animal was sacrificed. Therefore, the photos of Figure 3 are taken from different rabbits. Care was taken that all three rabbits in this study were of the same age and weight (as much as possible).

Table 1 shows the lumen area reduction obtained using histology, expressed as percentage with respect to the original size for the three arteries under examination (aorta, abdominal, and carotid), as a function of months elapsed after the initiation of the diet. The standard deviation of the average reduction in the lumen size is also provided. The reduction of lumen area with cholesterol diet at any month was compared with the lumen area without the diet. It was demonstrated that there is a significant reduction of the lumen area (*p* < 0.02, paired *t*-test, *n* = 3) with high cholesterol diet. A value of *p* > 0.05 was only observed when comparing months 4 and 3, indicating that after month 3, the lumen area is not reduced further.

**Table 1:** **Lumen reduction extracted from histology as percentage with respect to the original size for the three arteries under examination (aorta, abdominal, and carotid) as a function of month elapsed after the initiation of the diet**

*Aorta*	*N*	*Average reduction of area (%)*	*std*
*Month 0*	3	0	0
*Month 1*	3	15	4
*Month 2*	3	30	4.5
*Month 3*	3	48	9
*Month 4*	3	52	12
*Abdominal*	*N*	*Average reduction of area (%)*	*std*
*Month 0*	3	0	0
*Month 1*	3	12	3
*Month 2*	3	27	5.3
*Month 3*	3	43	8.3
*Month 4*	3	44	12
*Carotid*	*N*	*Average reduction of area (%)*	*std*
*Month 0*	3	0	0
*Month 1*	3	11	4
*Month 2*	3	22	2.5
*Month 3*	3	27	6.3
*Month 4*	3	32	8.8

The standard deviation of the average reduction in the lumen size is also provided.

**Figure 4:**
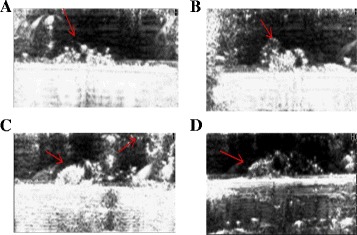
**Typical plaques in the rabbit carotid as imaged with ultrasound at 12 MHz.** In three of the cases, the reduction occurs in one side of the rabbit carotid artery **(A, B, D)**. In one case, the plaque appears on both sides of the artery **(C)**. Plaques are indicated with arrows.

Figure 4 shows typical plaques in the carotid artery as imaged with ultrasound at 12 MHz. In three of the cases, the reduction occurs in one side of the carotid artery. In one case the plaque appears on both sides of the carotid artery (Figure 4C).

**Figure 5:**
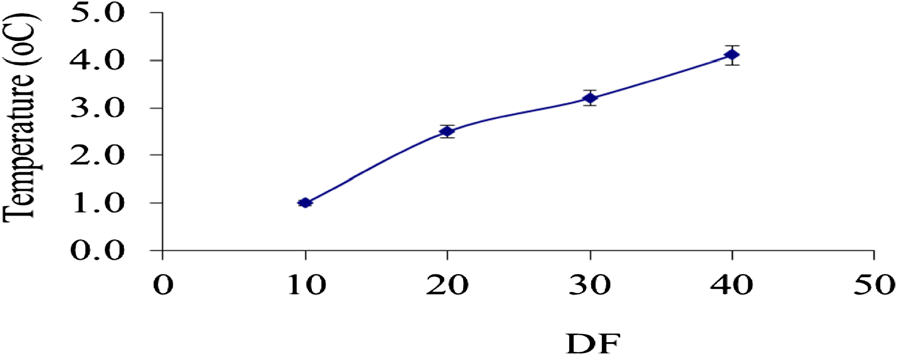
**Steady state temperature measured in the carotid with respect to DF with intensity of 30 W/cm** ^**2**^ **(SAPA), and PRF = 100 Hz.** Note that with DF above 10%, the temperature increases above 1°C and therefore some thermal effects are observed.

Figure 5 shows the steady state temperature measured with a thermocouple in the carotid with respect to duty factor (DF) with intensity of 30 W/cm^2^ (SAPA), and PRF = 100 Hz. Note that with DF greater than 10%, the steady state temperature increases above 1°C and therefore some thermal effects are observed.

**Figure 6:**
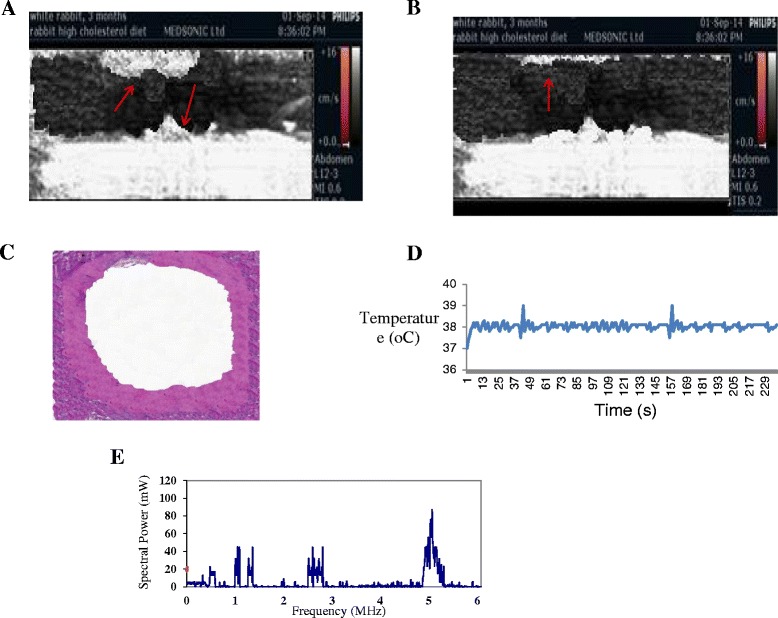
**Therapy results.** Ultrasonic image of the plaque in the carotid of the rabbit, photo of the HE staining of the carotid artery, temperature during plaque destruction, and frequency spectrum during the destruction of plaque. Ultrasonic image of plaque in the carotid of the rabbit with a plaque after 3 months of diet **(A)**. Ultrasonic image after the application of ultrasound with I = 30 W/cm^2^ (SAPA), PRF = 100 Hz, and DF = 10%. The plaque was removed after five sessions of 5-min injection of microbubbles **(B)**. Photo of the HE staining of the carotid artery showing small amount of residual plaque **(C)**. Temperature measured during the destruction of plaque, indicating temperature increase of approximately 1°C **(D)**. Frequency spectrum at one instance during the destruction of plaque indicating only subharmonic emissions which are indications of stable cavitation **(E)**.

Figure 6A shows the ultrasonic image of plaque in the carotid of the rabbit with a plaque after 3 months of diet. Figure 6B shows the ultrasonic image after the application of ultrasound with I = 30 W/cm^2^ (SAPA), PRF = 100 Hz, and DF = 10%. The plaque was removed after five sessions of 5-min injection of microbubbles. Note that almost all the plaque is removed. Figure 6C shows the photo of the HE staining of the carotid artery showing small amount of residual plaque. Figure 6D shows the temperature measured during the destruction of plaque, indicating temperature increase of approximately 1°C. Figure 6E shows the frequency spectrum at one instance, during the destruction of plaque, indicating only subharmonic emissions which are indicative of stable cavitation.

**Figure 7:**
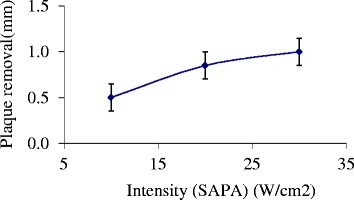
**Effect of intensity.** Size of the plaque removed as measured from the ultrasound images versus intensity (SAPA), D.F. = 10%, and PRF = 100 Hz.

The effect of the intensity on the removal of plaque was investigated. The carotid of three rabbits was exposed to different spatial average, pulse average (SAPA) intensity varying from 10 to 30 W/cm^2^, with the D.F. and PRF at 10% and 100 Hz, respectively. Figure 7 shows the size of the plaque removed, as measured from the ultrasound images, versus intensity (SAPA). During these experiments, the temperature did not exceed 1°C. The reduction of lumen size with the application of ultrasonic intensity at different levels was compared to the size of the untreated lumen. It was demonstrated that there is a significant reduction of the lumen size (p < 0.05, paired t-test, *n* = 3) with ultrasonic intensity.

## Conclusions

This paper includes a feasibility study that investigates the effectiveness of pulsed ultrasound in removing atherosclerotic plaque, created in rabbit with the final intention to remove atherosclerotic plaque from vessels. This feasibility study includes the effect of intensity and DF.

The experiments were conducted in the rabbit carotid with protocols leading to temperatures less than 1°C (safe temperature). It was demonstrated in a set of experiments that if the DF is increased, then the safe temperature is exceeded. Another parameter that increases the temperature is the intensity. In these experiments the intensity used was carefully chosen, and, therefore no temperature elevation above 1°C was produced. The removed plaque, as expected, increases with the intensity.

Very promising results were obtained in carrying out experiments in the carotid of rabbits. With this transducer and protocol we were able to remove atherosclerotic plaque up to a depth of 2 mm in 25 min. This type of technology looks promising for the removal of atherosclerotic plaque in humans, provided that the dissolved material from the plaque is collected. The collected material must not flow through the blood stream to other arteries, thus causing blockage of arteries. Care should be taken to collect all the removed particles. For example, in studies involving atherectomy [44,45], a suction mechanism was used to collect the removed particles. Such suction technology can also be incorporated with this ultrasound technology to remove the residual particles.

Also, it is possible that if the particle size is too small, then there is no need to collect the residual particles and, therefore, the deployment of ultrasound technology would be more feasible. Yet, clinical studies need to be done in order to reveal how small this residual particle must be. This specific device, with this size, can be used probably in peripheral arteries or in the carotid. Special design of the device needs to be done so as not to block the artery. For the application in the heart arteries, the device must be scaled down to possibly 1 mm.

Previously [40], therapeutic ultrasound was utilized using its thermal capabilities to ablate plaque in swine. With thermal ultrasound, plaques are heated and eventually destroyed. Due to the close proximity of the plaque to the artery, crucial thermal damaged tissue could be produced in the artery. With our method, pulse ultrasound is used in combination with microbubbles, and therefore plaque is detached leaving potentially no severe damage to the artery.

We clearly see the destruction of plaque using ultrasonic imaging, but what content of the plaque (lipid, calcium, or microphage) is destroyed first cannot be assessed accurately with ultrasound imaging.

The combination of pulsed ultrasound and microbubbles has been shown to be effective for removing clot [46] which is a much softer tissue than plaque. The use of ultrasound alone was ineffective to remove clot [46] in a rabbit carotid model. Microbubbles are known to cause stable cavitation and therefore assist the removal of clots. We speculated that the same effect (stable cavitation) could accelerate plaque removal. Further experiments need to be conducted to show that pulsed ultrasound alone will not be efficient in removing plaque as compared to pulsed ultrasound in synergy with microbubbles.

This technology can be used in the future for clinical trials primarily to treat plaques in the carotid. Unstable plaques in the carotid are a major source of plaques, which can reach the brain and cause stroke. The device can be attached to a catheter of appropriate size and the catheter can be guided intravenously to the carotid for ultrasonic treatment. Care will be taken to avoid the escape of debris reaching the brain, thus causing stroke.
